# MARPLE, a point-of-care, strain-level disease diagnostics and surveillance tool for complex fungal pathogens

**DOI:** 10.1186/s12915-019-0684-y

**Published:** 2019-08-13

**Authors:** Guru V. Radhakrishnan, Nicola M. Cook, Vanessa Bueno-Sancho, Clare M. Lewis, Antoine Persoons, Abel Debebe Mitiku, Matthew Heaton, Phoebe E. Davey, Bekele Abeyo, Yoseph Alemayehu, Ayele Badebo, Marla Barnett, Ruth Bryant, Jeron Chatelain, Xianming Chen, Suomeng Dong, Tina Henriksson, Sarah Holdgate, Annemarie F. Justesen, Jay Kalous, Zhensheng Kang, Szymon Laczny, Jean-Paul Legoff, Driecus Lesch, Tracy Richards, Harpinder S. Randhawa, Tine Thach, Meinan Wang, Mogens S. Hovmøller, David P. Hodson, Diane G. O. Saunders

**Affiliations:** 1 John Innes Centre, Norwich Research Park, Norwich, UK; 2 Ethiopian Institute of Agricultural Research, Addis Ababa, Ethiopia; 3 International Maize and Wheat Improvement Center (CIMMYT), Addis Ababa, Ethiopia; 4 Limagrain Cereal Seeds, 2040 SE Frontage Road, Fort Collins, CO 80525 USA; 5 RAGT Seeds Ltd, Cambridge, UK; 6 USDA-ARS and Department of Plant Pathology, Washington State University, Pullman, WA 99164 USA; 7 Nanjing Agricultural University, Nanjing, China; 8 Lantmännen Lantbruk, Svalöv, Sweden; 9 NIAB, Cambridge, UK; 10 Aarhus University Flakkebjerg, Slagelse, Denmark; 11 State Key Laboratory of Crop Stress Biology for Arid Areas and College of Plant Protection, Northwest A&F University, Yangling, 712100 Shaanxi China; 12 BASF Poland, Al. Jerozolimskie 142b, 02-305 Warsaw, Poland; 13 RAGT 2n, Centre de Recherche de Druelle, 12510 Olemps, France; 14 Sensako, Napier, Bethlehem, South Africa; 15 Agriculture and Agri-Food Canada, Lethbridge Research and Development Centre, Lethbridge, Alberta Canada

**Keywords:** Pathogen surveillance, Genomics, Point of care, Disease diagnostics, Wheat rust, Nanopore sequencing

## Abstract

**Background:**

Effective disease management depends on timely and accurate diagnosis to guide control measures. The capacity to distinguish between individuals in a pathogen population with specific properties such as fungicide resistance, toxin production and virulence profiles is often essential to inform disease management approaches. The genomics revolution has led to technologies that can rapidly produce high-resolution genotypic information to define individual variants of a pathogen species. However, their application to complex fungal pathogens has remained limited due to the frequent inability to culture these pathogens in the absence of their host and their large genome sizes.

**Results:**

Here, we describe the development of Mobile And Real-time PLant disEase (MARPLE) diagnostics, a portable, genomics-based, point-of-care approach specifically tailored to identify individual strains of complex fungal plant pathogens. We used targeted sequencing to overcome limitations associated with the size of fungal genomes and their often obligately biotrophic nature. Focusing on the wheat yellow rust pathogen, *Puccinia striiformis* f.sp. *tritici* (*Pst*), we demonstrate that our approach can be used to rapidly define individual strains, assign strains to distinct genetic lineages that have been shown to correlate tightly with their virulence profiles and monitor genes of importance.

**Conclusions:**

MARPLE diagnostics enables rapid identification of individual pathogen strains and has the potential to monitor those with specific properties such as fungicide resistance directly from field-collected infected plant tissue in situ. Generating results within 48 h of field sampling, this new strategy has far-reaching implications for tracking plant health threats.

**Electronic supplementary material:**

The online version of this article (10.1186/s12915-019-0684-y) contains supplementary material, which is available to authorized users.

## Background

Rapid and accurate point-of-care (PoC) diagnostics facilitate early intervention during plant disease outbreaks and enable disease management decisions that limit the spread of plant health threats. PoC diagnostics involve portable equipment that can be used in-field to rapidly confirm disease outbreaks and provide actionable information [1]. At present, conventional plant disease diagnostics rely on visible inspections of disease symptoms followed by basic laboratory tests through culturing and pathogenicity assays [2]. Unfortunately, these conventional methods tend to be subjective, time-consuming, labour-intensive and reliant on specialised expertise and equipment, providing only limited phenotypic information [3]. These factors limit their utility in PoC diagnosis.

Recent alternative approaches have focused on serological and nucleic acid assays [4]. Polyclonal and monoclonal antisera are frequently used to detect plant pathogens using techniques such as enzyme-linked immunosorbent assay (ELISA), immunostrip assays and immunoblotting [5]. In addition, following a flurry of PCR-based diagnostic tests in the 1980s, the advent of the loop-mediated isothermal amplification (LAMP) assay at the turn of the twenty-first century provided the first rapid nucleic acid amplification method to accurately diagnose pathogens in situ in real time [6]. Both serological and DNA-based methods typically require high initial financial investments and specialised expertise to develop new assays, are limited in sample capacity, frequently are not reliable at the asymptomatic stage, and provide limited information beyond the species level [1].

The capacity to distinguish between individuals in a pathogen population with specific properties such as fungicide resistance, toxin production and virulence profiles is often essential to inform disease management approaches. In the past two decades, the genomics revolution has led to technologies that can rapidly generate genome-scale genetic information to define individual variants of a pathogen species [4]. These emerging, data-driven, PoC diagnostic tools have the potential to rapidly track shifting pathogen populations in near real-time, providing copious genetic information at the strain level that can be used in early warning systems and disease forecasting.

The value of portable genomic-based diagnostics and surveillance was first illustrated during emergent human health outbreaks. For instance, during the Ebola crisis in West Africa in 2015, genome sequencing of the virus was carried out in situ on the first portable genome sequencer, the Oxford Nanopore Technologies MinION sequencer [7]*.* The resulting real-time genomic information on evolutionary rates and epidemiological trends revealed frequent transmission across the Guinea border [7], which informed subsequent disease control measures. For plant diseases, a similar approach in the laboratory environment successfully identified *Plum pox virus* and ‘*Candidatus* Liberibacter asiaticus’, which causes citrus greening in infected insect and plant tissues [8], exemplifying the potential for the development of portable genomic-based diagnostics for plant health threats. However, for higher-order fungal pathogens which constitute the largest and most widely dispersed group of plant pathogens [9], the utility of mobile genomic-based PoC diagnostics remains to be fully realised. The sheer size of fungal genomes, which can be tens or even hundreds of thousands of times larger than viral genomes, makes full-genome or whole-transcriptome sequencing on portable sequencing devices currently prohibitively expensive.

In this study, we developed an approach for generating high-throughput sequencing data in situ from the complex obligately biotrophic fungal pathogen *Puccinia striiformis* f. sp. *tritici* (*Pst*). *Pst* is a basidiomycete and heterokaryotic fungus that causes wheat yellow rust disease, which is a constant and significant threat to wheat production worldwide [10]. We demonstrate herein that our approach can be used to rapidly define individual *Pst* strains, assign strains to distinct genetic lineages that have been shown to correlate tightly with their virulence profiles [11], and monitor mutations in genes of importance. As *Pst* is an obligate biotroph, the genetic material of the pathogen and plant have to be analysed together in field-collected infected samples. Furthermore, the pathogen’s genome is more than 10,000 times larger than that of, for instance, the Ebola virus.

To address these complexities, we first utilised a comparative genomics approach to define genomic regions of high variability between pathogen strains that could then be amplified for sequencing directly from field-infected wheat samples on the mobile nanopore sequencer. This new approach thereby circumvents the need to carry out lengthy in-lab processes of purification and multiplication of isolates prior to high molecular weight DNA extraction that is a requirement for full genome sequencing. This targeted sequencing approach also reduced the complexity and amount of data generated per sample, thereby accelerating the speed of processing and reducing the cost. Furthermore, we developed a mobile lab system to enable deployment of our diagnostic platform in resource-poor regions without the need for continuous electricity or access to additional laboratory equipment. This Mobile And Real-time PLant disEase (MARPLE) diagnostics system was designed with simplicity and mobility in mind to enable true PoC plant disease diagnostics. This new strategy has the potential to revolutionise plant disease diagnostics, changing how plant health threats are identified and tracked into the future.

## Results

### Capturing the global diversity of the *Pst* population

To reduce the complexity of the genomic data generated from *Pst*-infected wheat samples, we aimed to define regions of the *Pst* genome showing high variability between pathogen strains that could be subsequently amplified for sequencing on the MinION platform from Oxford Nanopore Technologies. The first step was to capture the diversity of the *Pst* global population. To achieve this, we carried out transcriptome sequencing on 100 *Pst*-infected wheat samples collected between 2015 and 2017 from nine countries, including those in eastern and southern Africa, Europe, North America and Asia (Additional file 1: Table S1). Total RNA was extracted from each sample and subjected to RNA sequencing (RNA-seq) analysis using the Illumina HiSeq platform and our previously described field pathogenomics strategy [11]. To maximise the geographical distribution of *Pst* isolates, we combined these 100 RNA-seq datasets with previously published genomic and transcriptomic datasets from a further 201 *Pst* strains spanning a total of 19 countries, including Chile, New Zealand, Pakistan and an array of European countries [11, 12] (Additional file 1: Table S1). Raw reads were filtered for quality, and data from each *Pst* sample were independently aligned to the *Pst* race PST-130 reference genome [13]. An average of 37.3% (± 18.2%, S.D.) reads aligned to the reference genome for the combined RNA-seq datasets, and 82.7% (± 4.9%, S.D.) reads aligned for the genomic datasets [11] (Additional file 1: Tables S2 and S3). Overall, the data from this global collection of *Pst* isolates comprised 280 transcriptomic and 21 genomic datasets from *Pst* isolates spanning 24 countries that could then be used for subsequent population genetic analysis.

**Fig. 1:**
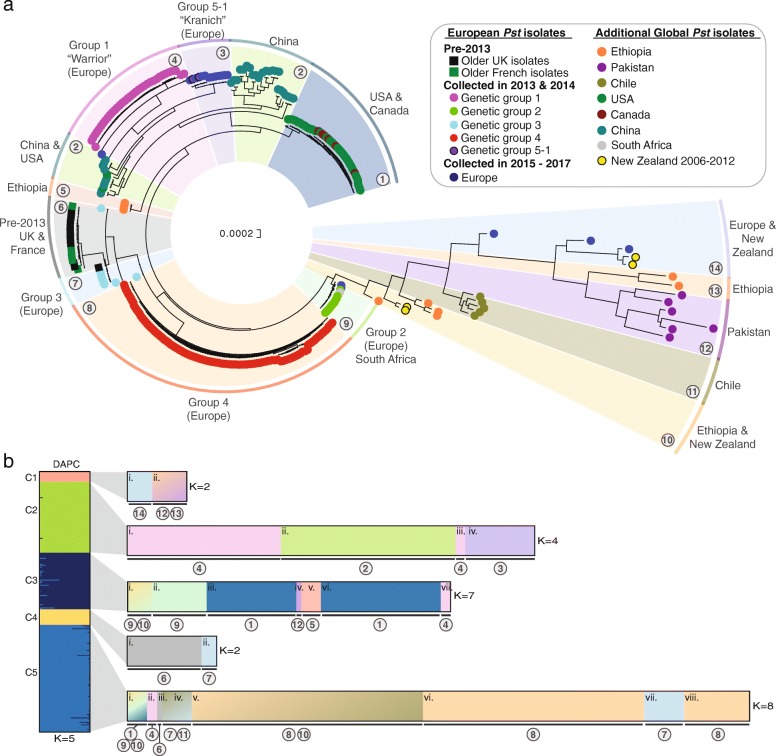
The global *Pst* population is highly diverse and largely consists of geographically isolated groups of distinct homogenous individuals. **a** The global *Pst* population analysed herein consisted of 14 distinct groups of individuals. Phylogenetic analysis was performed on a total of 280 transcriptomic and 21 genomic datasets from *Pst* isolates spanning 24 countries, using a maximum-likelihood model and 100 bootstraps. Scale indicates the mean number of nucleotide substitutions per site. Bootstrap values are provided in Additional file 3. **b** Multivariate discriminant analysis of principal components (DAPC) could further define subdivisions within the global *Pst* population. A list of 135,139 biallelic synonymous single nucleotide polymorphisms (SNPs) was used for DAPC analysis. Assessment of the Bayesian Information Criterion (BIC) supported initial division of the *Pst* isolates into five genetically related groups (left; C1–5). Due to the high level of diversity among the global *Pst* population, this initial analysis could not resolve *Pst* isolates with lower levels of within-group variation. Therefore, a second DAPC analysis was carried out on each of the five initial population groups (right). Bar charts represent DAPC analysis, with each bar representing estimated membership fractions for each individual. Roman numerals represent the successive *K* values for each DAPC analysis. Numbers in circles are reflective of those assigned to distinct groups in the phylogenetic analysis

To determine the genetic relationships between these 301 global *Pst* samples, we carried out phylogenetic analysis using the third codon position of 2034 PST-130 gene models (589,519 sites) using a maximum-likelihood model (Additional files 2 and 3). *Pst* isolates tended to cluster based on their geographical origin, with only four of the 14 divisions containing *Pst* isolates that spanned continental boundaries (Fig. 1a). These four clades included (i) clade 2 with *Pst* isolates from China and the USA, (ii) clade 9 with *Pst* isolates from Europe and South Africa, (iii) clade 10 containing *Pst* isolates from Ethiopia and New Zealand and (iv) clade 14 containing isolates from Europe and New Zealand (Fig. 1a). This represents relatively recent shared ancestry between the populations within these four clades, which could be indicative of long-distance transmission of *Pst* strains either between these regions or from a common independent source area.

We carried out multivariate discriminant analysis of principal components (DAPC) to further define subdivisions within the global *Pst* population. First, we generated a list of 135,372 synonymous single nucleotide polymorphisms (SNPs), of which 135,139 were biallelic in at least one *Pst* sample and were therefore used for DAPC analysis. Assessment of the Bayesian Information Criterion (BIC) supported division of the *Pst* isolates into five groups of genetically related *Pst* isolates (Additional file 4). However, due to the high level of diversity within the global *Pst* population, this initial DAPC analysis was able to separate only *Pst* populations with high levels of genetic differentiation and was unable to resolve lower levels of within-group variation [14] (Fig. 1b). For instance, group 1 (C1) contained *Pst* isolates from Pakistan, Ethiopia, Europe and New Zealand, and group 2 (C2) contained *Pst* isolates from China and two European races that have been shown to be genetically distinct in previous population studies [12, 15]. Therefore, we performed further DAPC analysis on each of the five population groups independently and, following analysis of the BIC, *Pst* isolates were separated into clear subsets of homogenous groups of individuals that better reflected the phylogenetic clustering (Fig. 1b; Additional file 4). Overall, this analysis indicated that the global *Pst* population is highly diverse and, with only a few exceptions, consists of geographically isolated groups of distinct homogenous individuals.

### A subset of genes can be used to capture the global diversity of *Pst* isolates

To identify specific *Pst* genes contributing to the separation of isolates into distinct groups in the population genetic analysis, we used comparative analysis to find the most variable genes among the 301 global *Pst* isolates that were conserved across all *Pst* isolates analysed. First, we calculated the number of SNPs per kilobase for each gene from alignments of sequences representing the 301 *Pst* isolates against the PST-130 reference genome [13]. SNPs per kilobase values were calculated by normalising the total number of SNPs found in the coding sequence of each gene across the 301 *Pst* isolates relative to the length of the coding sequence for each gene. A total of 1690 genes were identified as polymorphic (SNPs/kb ≥ 0.001) between *Pst* isolates and subsequently utilised for phylogenetic analysis with a maximum-likelihood model. Importantly, the sequences from these 1690 polymorphic genes were sufficient to reconstruct the topology of the global *Pst* phylogeny (Additional file 5).

**Fig. 2:**
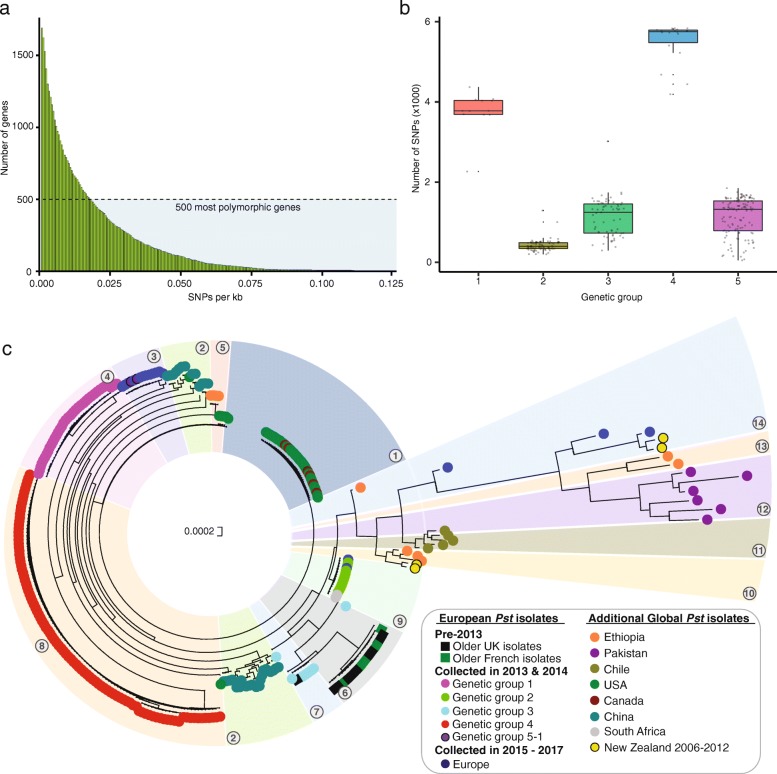
The sequences of 242 highly polymorphic *Pst* genes are sufficient to reconstruct the topology of the global phylogeny generated from full transcriptome and genome sequencing**. a** Ordered distribution of average SNP content per gene across the 301 *Pst* global isolates. To determine the minimum number of gene sequences required to accurately reconstruct the global phylogeny, the 1690 genes identified as polymorphic (SNPs/kb ≥ 0.001) between *Pst* isolates were ordered according to number of polymorphic sites across the 301 global *Pst* isolates. **b** The 242 polymorphic genes selected were not biased in their selection by a high degree of divergence from the reference race PST-130 for any particular group of individuals. Box plots represent the total number of SNPs across these 242 genes for *Pst* isolates belonging to each of the five major genetic groups identified through DAPC analysis. Bar represents median value, box signifies the upper (Q3) and lower (Q1) quartiles, data falling outside the Q1–Q3 range are plotted as outliers. **c** The 242 genes selected could be used successfully to reconstruct the global phylogeny and assign *Pst* isolates to the 14 previously defined groups (numbers in circles). Phylogenetic analysis was performed using sequence data for the 242 genes from the 301 global *Pst* isolates using a maximum-likelihood model and 100 bootstraps. Bootstrap values are provided in Additional file 7

To determine the minimum number of gene sequences required to accurately reconstruct the global phylogeny, we ordered the 1690 genes based on the number of polymorphic sites across the 301 *Pst* isolates (Fig. 2a). We then selected 1006, 748, 500, 402, 301, 204, 151 and 100 of the most polymorphic genes using progressively increasing cut-off values for SNPs per kilobase (0.006, 0.0105, 0.018, 0.023, 0.0285, 0.036, 0.042 and 0.051, respectively) and carried out phylogenetic analysis as described above with each of these subsets (Additional file 5). We noted that a single *Pst* isolate from clade 9 was mis-assigned to clade 4 in the phylogenies reconstructed from fewer than 500 genes (Additional file 5). This inconsistency was likely due to poor gene coverage for this *Pst* isolate when the data were aligned to the PST-130 reference genome; for instance, 96.5% of bases had less than 20× coverage when using 402 *Pst* genes to reconstruct the phylogeny. Therefore, this *Pst* isolate (14.0115) was excluded from the general evaluation. Overall, we concluded that whilst minor changes in clade ordering were observed when using sequence data from less than 500 genes, sequence data from as few as 100 genes were sufficient to generate a similar phylogeny topology (Additional file 5) and assign *Pst* isolates to the 14 previously defined groups.

The next step was to use the minimal number of polymorphic genes required to represent *Pst* population diversity to define a subset of genes for PCR amplification in preparation for sequencing on the MinION platform. We reasoned that sequencing a small subset of highly variable genes would reduce the volume of data generated and associated cost per sample, whilst maintaining our ability to define individual strains. We selected the 500 most polymorphic genes between *Pst* isolates and within this subset randomly selected 250 of these genes; oligonucleotides were successfully designed for 242 genes (Additional file 1: Table S4). Given that a minimum of 100 genes was sufficient to accurately assign *Pst* isolates, the additional 142 genes were included to ensure that *Pst* isolates could be correctly assigned even if a large proportion (up to 58%) of the genes failed to amplify under field conditions. To validate that the 242 polymorphic genes were not biased in their selection by a high degree of divergence from the reference isolate PST-130 for any particular group of individuals, we assessed the total number of SNPs across these 242 genes for *Pst* isolates belonging to each of the five major genetic groups identified through DAPC analysis (Fig. 1b). The SNPs were distributed across all the major genetic groups, with the least number of SNPs identified in *Pst* isolates of genetic group 2 and the greatest number identified in *Pst* isolates from genetic group 4 (Fig. 2b). The low differentiation of *Pst* isolates in genetic group 2 from the PST-130 reference isolate likely reflects a close genetic relationship. Finally, we confirmed that the 242 genes selected could be used successfully to reconstruct the global phylogeny and assign *Pst* isolates to the 14 previously defined groups (Fig. 2c; Additional files 6 and 7). Overall, this analysis illustrated that using sequence data from a minimal set of 242 polymorphic *Pst* genes was sufficient to accurately genotype *Pst* isolates and re-construct a comparable phylogeny to that achieved from full-genome or transcriptome sequencing.

### Genes selected for amplicon sequencing are distributed across the *Pst* genome and the majority encode enzymes

**Fig. 3:**
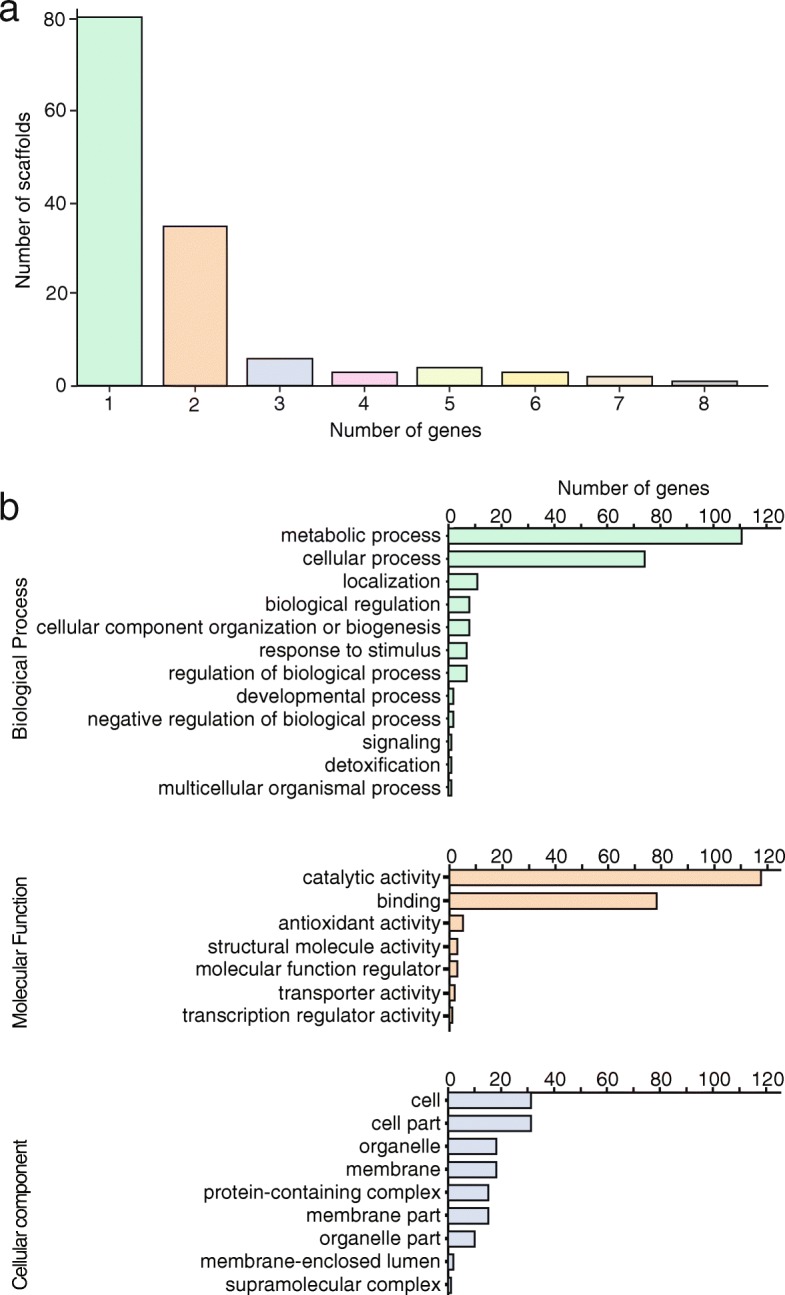
The 242 *Pst* genes selected are evenly distributed across the *Pst* genome and a large proportion encode proteins with enzymatic functions. **a** For 241 of the 242 genes, near-identical (> 94% pairwise identity) hits were identified in the more contiguous *Pst*-104 genome and 60% were located on scaffolds that contained only one of the 241 genes. Bar chart illustrates the number of genes identified on the given numbers of scaffolds. **b** Functional annotation of the 242 *Pst* genes selected for MinION sequencing revealed that they largely encode proteins with enzymatic functions. Bar charts illustrate GO term analysis, with gene functions associated with ‘Biological process’, ‘Metabolic function’ and ‘Cellular component’ highlighted

To characterise the 242 *Pst* genes selected for sequencing on the MinION platform, we carried out positional and functional annotation. To assess the distribution of the 242 polymorphic genes across the *Pst* genome, we identified their genomic locations in the highly contiguous *Pst*-104 reference genome [16]. For 241 of the 242 genes, near-identical (> 94% pairwise identity) hits in the genome were obtained when gene sequences were mapped to the genome using minimap2 [17]. These 241 genes were distributed across a total of 135 genome scaffolds, with the majority of genes (60%) located on scaffolds that contained only one of the 241 genes (Additional file 1: Table S5). Only 10 scaffolds contained more than five of these genes, suggesting that the majority of the 241 genes were scattered across the genome and not grouped in gene clusters (Fig. 3a). Using gene ontology (GO) term analysis, we found that the majority (64%) of the 242 genes encoded proteins with enzymatic functions (GO: 0003824—catalytic activity; GO: 0005488—binding) and were involved in different metabolic and cellular processes (Fig. 3b; Additional file 1: Table S5). Overall, this analysis indicates that 241 of the 242 *Pst* genes selected are well distributed across the *Pst* genome and are enriched for functions in fungal metabolism.

### Comparative analysis of the Illumina and Oxford Nanopore sequencing platforms

To assess the suitability of the mobile MinION sequencer for population diversity analysis using the 242 *Pst* genes selected, we carried out a comparative analysis with data generated on the Illumina MiSeq platform, which is frequently used for this purpose [18]. Four *Pst*-infected wheat samples were collected in 2017 in Ethiopia (Additional file 1: Table S1). Following genomic DNA extraction, each of the aforementioned 242 *Pst* genes was amplified from each sample. Each gene was then used for amplicon sequencing on both the MinION and MiSeq platforms. A total of 6.9, 3.6, 6.2 and 6.4 million paired-end Illumina reads and 109, 102, 128 and 113 thousand MinION reads were generated for each of the four *Pst*-infected wheat samples (17.0504, 17.0505, 17.0506 and 17.0507 respectively). Following base calling and quality filtering, reads were aligned to the gene sequences for the 242 genes from the PST-130 reference [13] (Additional file 1: Table S6 and S7). For each *Pst*-infected sample, consensus sequences were generated for each of the 242 genes, using data produced on the Illumina MiSeq platform. Each consensus gene set separately incorporated the SNPs identified within the gene space by mapping the reads from each of the four *Pst* isolates against the gene sequences of the 242 genes. These four sets of sequences formed an accurate baseline for comparison with sequence data generated on the MinION sequencer.

**Fig. 4:**
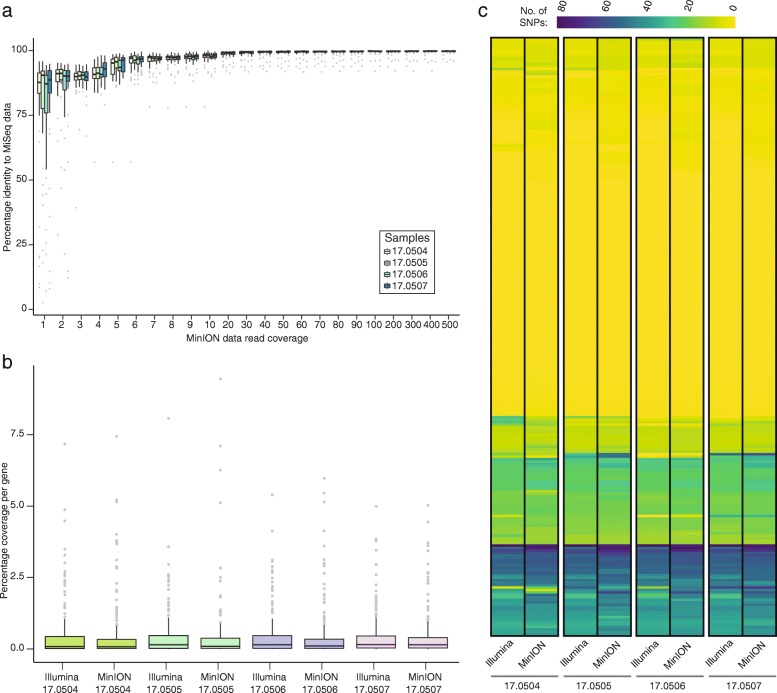
A minimum of 20x depth of coverage on the MinION sequencer is sufficient to generate comparable gene sequence data to the Illumina MiSeq platform**. a** At 20x coverage on the MinION sequencer, comparisons with data generated on the Illumina MiSeq platform showed 98.74% sequence identity. **b** No notable selective bias occurred during library preparation and sequencing of individual genes using either the MiSeq or MinION platforms. Box plots show the percentage coverage for each of the 242 *Pst* genes sequenced for the four *Pst* isolates tested on the MinION and MiSeq platforms. **c** The number of SNPs per gene detected in each of the four MinION datasets was comparable to that from the MiSeq platform. Heatmaps represent the number of SNPs identified per gene (*y*-axis) for the four *Pst* isolates sequenced on the MinION and MiSeq platforms. Full details regarding the number of SNPs identified per gene are provided in Additional file 1: Table S9. In box plots **a** and **b**, bars represent median value, boxes signify the upper (Q3) and lower (Q1) quartiles, data falling outside the Q1–Q3 range are plotted as outliers

To evaluate the minimum depth of coverage required to obtain similar levels of accuracy on the MinION sequencer, we performed a comparative analysis between the two platforms. Sequence data generated on the MinION platform were used to create consensus sequences for each of the aforementioned 242 *Pst* genes using varying depths of coverage for each of the four *Pst*-infected wheat samples. The percentage identity of these consensus sequences was then determined through comparative analysis with the MiSeq baseline consensus sequences. A minimum depth of 20× coverage on the MinION sequencer was sufficient to achieve 98.74% sequence identity between the two datasets (Fig. 4a).

We then investigated whether there was any notable selective bias during library preparation and sequencing of individual genes using either the MiSeq or MinION platform. We determined the percentage coverage for each of the 242 genes sequenced for the four *Pst* isolates on the two sequencing platforms. The average coverage per gene for the MiSeq (0.41 ± 0.02, S.E.) and MinION (0.41 ± 0.03, S.E.) platforms was comparable (Fig. 4b).

Using the predefined 20× coverage level, we evaluated the required run time to achieve this level of coverage across all 242 selected *Pst* genes on the MinION platform. Assuming equal coverage of all genes, we determined that to reach 20× coverage for all 242 genes in each of the four samples (4840 reads) would take less than 30 min from starting the MinION sequencing run [18.75 (17.0504), 21.77 (17.0505), 17.65 (17.0506) and 19.20 (17.0507) minutes] (Additional file 1: Table S8).

Finally, using the minimum level of 20× depth of coverage for data generated on the MinION sequencer, we defined the number of SNPs per gene in each of the four MinION datasets. This was then compared with SNP analysis using sequence data generated on the MiSeq platform. SNP profiles for each of the samples sequenced on the MinION and the MiSeq platforms were largely comparable, with the general trend being that more SNPs (compared with the reference) were identified when sequencing was carried out on the MinION platform (Fig. 4c; Additional file 1: Table S9). In particular, we observed that several positions that were designated as being homokaryotic from data generated on the MiSeq platform appeared as heterokaryotic when using the MinION sequencer. The average ratio of heterokaryotic to homokaryotic nucleotide positions using the MiSeq platform was 0.01 (± 0.0002, S.D.), which was 20% higher (0.012 ± 0.0004, S.D.) when the MinION sequencer was used (Additional file 1: Table S10). However, as the overall average sequence identity between samples sequenced using the MiSeq and MinION platforms was > 98%, we concluded that when a minimum of 20× depth of coverage is achieved, the data generated on the MinION sequencer are largely comparable in accuracy to those from the MiSeq platform and therefore should be suitable for population genetic analysis.

### *Pst* isolates from Ethiopia in the 2017/2018 wheat crop season are genetically closely related

To further assess the ability of the MinION-based sequencing platform to accurately define *Pst* genotypes in field-collected infected samples, we expanded our analysis to a larger sample of 51 *Pst*-infected wheat samples collected in Ethiopia predominantly during the 2017/2018 growing season (Additional file 1: Table S1). DNA was extracted from each sample independently, and each of the aforementioned 242 *Pst* genes was amplified and prepared for amplicon sequencing on the MinION platform. In parallel, RNA was extracted and RNA-seq analysis was undertaken using the Illumina HiSeq platform and our field pathogenomics strategy for comparison [11]. An average of 114,519.37 (± 91,448.42, S.D.) reads per library were generated using the MinION sequencer and a total of 23,434,565.49 (± 2,468,438.63, S.D.) reads per library were generated on the HiSeq platform (Additional file 1: Tables S7 and S11). Following base calling and data filtering, reads generated on the HiSeq or MinION platforms were aligned independently for the 51 *Pst* isolates to sequences of the 242 *Pst* genes selected.

**Fig. 5:**
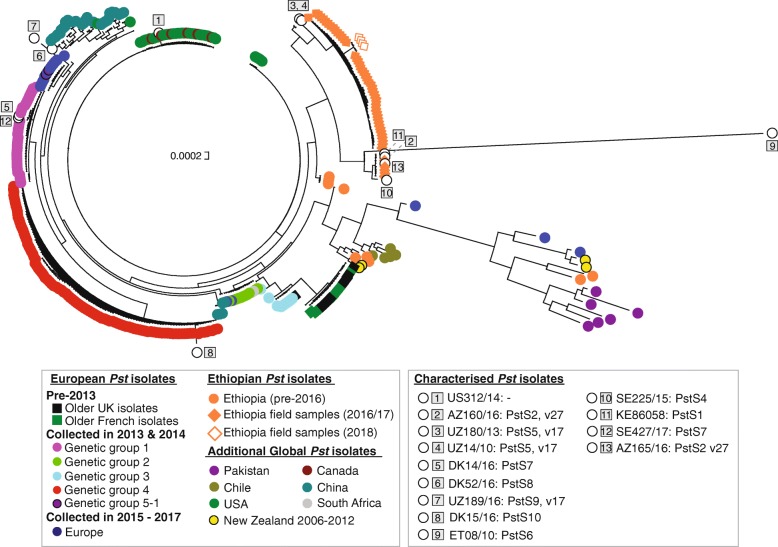
Gene sequencing on the MinION platform can be used to accurately genotype *Pst* isolates and define specific race groups**.** All Ethiopian *Pst* isolates collected from 2016 onwards cluster in a single monophyletic group (orange diamonds). The 13 representatives of previously defined race groups (numbered squares) tended to cluster in the phylogeny with *Pst* isolates of a similar genetic background. Phylogenetic analysis was carried out using a maximum-likelihood model and 100 bootstraps. Scale indicates the mean number of nucleotide substitutions per site. Bootstrap values are provided in Additional file 10

We then carried out the phylogenetic analysis as described above, using data from either the MinION or HiSeq platforms independently (Fig. 5; Additional files 8, 9, 10, 11 and 12). To compare the Ethiopian *Pst* isolates with the global *Pst* population groups, we also included sequence data for the 242 genes from the 301 global *Pst* isolates in the phylogenetic analysis. The positioning of the 51 Ethiopian samples in the phylogenies was similar between the two datasets, with the 51 *Pst* field isolates grouping in two closely related clades in both cases (Fig. 5 and Additional file 8). This analysis further supports the conclusion that when a sufficient level of coverage is used, data generated on the MinION platform can be used to accurately define *Pst* genotypes.

### Assigning *Pst* isolates to known genetic groups defined by SSR marker analysis

To compare the phylogenetic clades with previously defined *Pst* genetic groups based on simple sequence repeat (SSR) marker analysis and pathogenicity testing [15, 19–21], we selected 13 additional *Pst* isolates of diverse origin representing these groups (Additional file 1: Table S12). DNA was extracted from each sample independently, and the 242 *Pst* genes were amplified and prepared for sequencing on the MinION platform. Following base calling and quality filtering, reads were aligned to sequences of the 242 PST-130 genes. The resulting data were then combined with those from the 301 global *Pst* isolates and the 51 Ethiopian *Pst* isolates collected predominantly during the 2017/2018 field season, and phylogenetic analysis was performed (Fig. 5; Additional files 9 and 10).

The 13 *Pst* isolates representing previously defined *Pst* groups and races clustered in the phylogeny as follows. US312/14 (a.k.a AR13–06), representing a new group of isolates in North America carrying virulence to the yellow rust (Yr) resistance gene *Yr17*, grouped in a clade with other recent *Pst* isolates that were collected in the USA and Canada in 2015 and 2016. AZ160/16 and AZ165/16 belonging to the *PstS2*, v27 group, which has been prevalent in eastern and northern Africa and western Asia, grouped with *Pst* isolates from Ethiopia. UZ180/13 and UZ14/10, both representing the *PstS5*, v17 group prevalent in central Asia, was basal to a clade of Ethiopian *Pst* isolates. UZ189/16 (*PstS9*, v17), frequently found in central Asia, formed a distinct branch in the phylogeny. ET08/10, representative of the *PstS6* group and carrying virulence to *Yr27*, formed a long unique branch. SE225/15, which belongs to the *PstS4* race (a.k.a. ‘Triticale2006’) and is frequently found on triticale in Europe, formed a distinct branch close to *Pst* isolates from Ethiopia. KE86058, a representative of the *PstS1* aggressive strain recovered from the ‘Stubbs Collection’, grouped with isolates from Ethiopia. DK14/16 and SE427/17 representing the ‘Warrior’ *PstS7* group, DK52/16 representing the ‘Kranich’ *PstS8* group and DK15/16 representing the ‘Warrior(-)’ *PstS10* group, shown to be analogous to ‘genetic group 1’, ‘genetic group 5-1’ and ‘genetic group 4’, respectively [12], clustered accordingly in the phylogeny (Fig. 5). This result illustrates that data generated on the MinION platform for the 242 polymorphic *Pst* genes can be used to accurately distinguish the genetic groups previously defined from SSR marker-based classification, providing additional support to the methodology herein. Furthermore, the inclusion of these reference *Pst* isolates in future analysis will enable isolates of similar genetic background to be rapidly identified.

### In-field MinION-based diagnostics can define *Pst* isolates in Ethiopia in real-time

**Fig. 6:**
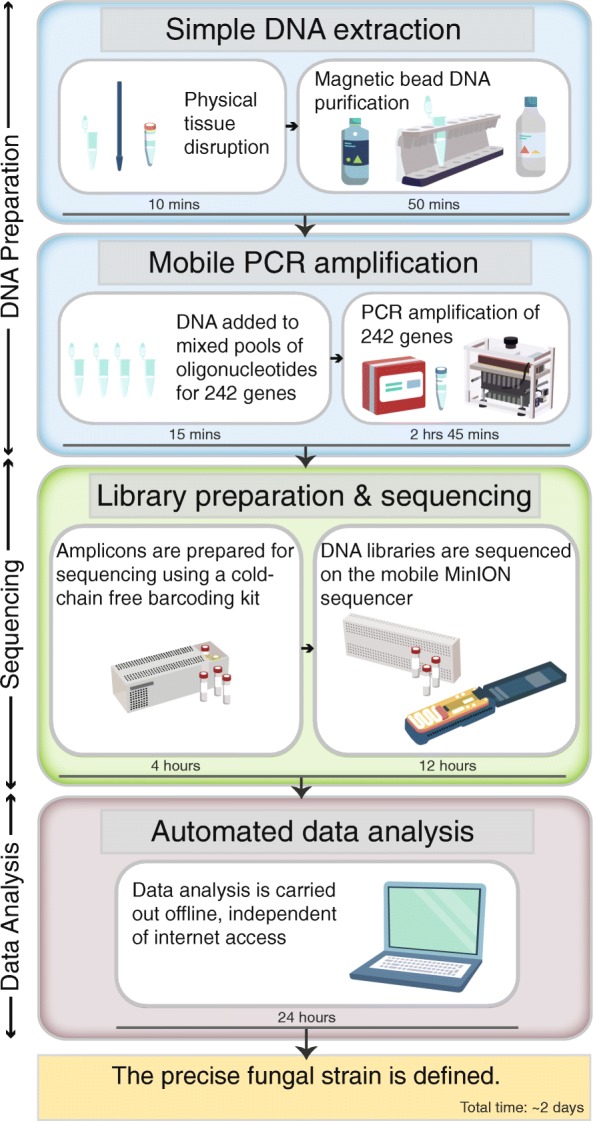
Illustration of the MARPLE pipeline**.** A simplistic Mobile And Real-time PLant disEase (MARPLE) diagnostics pipeline was developed so that the 242 polymorphic *Pst* genes could be amplified and sequenced on the MinION platform for population genetic analysis in situ*.* This pipeline consists of three stages (DNA preparation, Sequencing and Data analysis) and can be executed independently of stable electricity or internet connectivity in less than 2 days from sample collection to completion of the phylogenetic analysis

As resource-poor locations frequently bear the brunt of plant disease epidemics, we developed a simplistic Mobile And Real-time PLant disEase (MARPLE) diagnostics pipeline so that the 242 polymorphic *Pst* genes could be amplified and sequenced on the MinION sequencer for phylogenetic analysis in situ (Fig. 6; Additional file 13). To test our MARPLE diagnostics pipeline, we collected four *Pst*-infected wheat samples in 2018 and carried out analysis in situ in Ethiopia (Additional file 1: Table S1). As Ethiopia may act as a gateway for new *Pst* isolates entering Africa from sexually recombining populations in Asia, this pipeline would enable rapid detection of any new *Pst* strains entering East Africa.

First, DNA was extracted from each *Pst*-infected wheat sample using a simplified method wherein *Pst*-infected plant tissue was homogenised, cells lysed and DNA isolated using magnetic-bead-based purification (Fig. 6). Next, a panel of 242 oligonucleotide pairs was used in PCR amplification to enrich for the previously defined set of *Pst* gene sequences. This enrichment enabled direct analysis of each of the field-collected *Pst*-infected wheat tissue samples. The 242 oligonucleotide pairs were pooled into four groups, where concentrations were optimised individually to amplify all genes in each individual group (Additional file 1: Table S4). To ensure ease of portability and avoid the need for continuous electricity, PCR amplification was performed using thermostable *Taq* polymerase and a battery-powered mobile miniPCR machine (Fig. 6; Additional file 1: Table S13). Finally, a simple analysis pipeline independent of internet connectivity was utilised on a laptop computer for phylogenetic analysis of *Pst* isolates (Fig. 6).

Overall, the entire pipeline from sample collection to completion of the phylogenetic analysis was achieved within 2 days, providing rapid real-time information on the population dynamics of *Pst* in Ethiopia. The resulting phylogenetic analysis of the four *Pst*-infected wheat samples illustrated that the late 2018 *Pst* population in Ethiopia was similar to that defined in the previous 2017/2018 growing season (Fig. 5).

## Discussion

### Utility of mobile gene sequencing for plant pathogen surveillance

Effective disease management depends on timely and accurate diagnosis that can be used to guide appropriate disease control decisions. For many plant pathogens, including *Pst*, visual inspection at the symptomatic stage provides clearly recognisable indications of the causative agent. However, the ability to go beyond visual species-level diagnostics and rapidly define newly emergent strains or identify those with specific properties such as fungicide resistance, toxin production or specific virulence profiles (races) helps tailor proportionate and effective disease control measures. For most fungal plant pathogens, diagnostic methods providing strain-level resolution remain highly dependent on time-consuming and costly controlled bioassays carried out by specialised laboratories. However, the genomic revolution has provided opportunities to explore rapid strain-level diagnostics. The advent of mobile sequencing platforms allows these systems to become geographically flexible and independent of highly specialised expertise and costly infrastructure investment.

Here, we used the mobile MinION sequencing platform to develop a genomic-based method called MARPLE diagnostics for near real-time PoC plant disease diagnostics for fungal pathogens, which have proved less tractable for such approaches. The size of fungal genomes makes full-genome or transcriptome sequencing on portable devices prohibitively expensive. Furthermore, for at least the wheat rust pathogens the lengthy processes associated with purification and multiplication of isolates for high molecular weight DNA extraction has prevented whole genome sequencing being used for PoC strain-level diagnostics. By focusing on sequencing 242 highly variable genes that are informative for distinguishing individual *Pst* lineages, we were able to reduce the volume of data required whilst maintaining the ability to define individual strains. Analysis of this highly polymorphic gene set revealed it to be rich in genes with functions in fungal metabolism, with a large number of these genes encoding enzymes.

The approach we have taken herein is extremely flexible, and the existing gene panel can be easily supplemented with additional genes as required. For instance, as avirulence proteins that trigger host immune responses are identified in *Pst*, the corresponding genes can be incorporated into the method and monitored for mutations that could be linked to a gain of virulence. Furthermore, including genes that encode proteins identified as conserved fungicide targets across fungal pathogens would be extremely valuable. This would enable real-time monitoring of known mutations that have been linked to decreases in sensitivity in other pathosystems. For the wheat rust pathogens, the two main classes of fungicides at risk of resistance developing are the triazole demethylation inhibitors that target the *cyp51* gene and the succinate dehydrogenase (SDH) inhibitors that target genes encoding the four subunits of the SDH complex [22]. Incorporation of *cyp51* and the four SDH complex genes in the gene panel for *Pst* is currently underway and will provide real-time monitoring that can rapidly detect any novel mutations as they emerge, ensuring chemical control strategies are modified accordingly. The incorporation of high-resolution genomic data into clinical diagnostics and surveillance for human health has demonstrated the utility of such approaches in rapidly identifying drug-resistance mutations, accurately typing strains and characterising virulence factors [23]. The integration of such rapid genomic-based diagnostic data enables detection and appropriate action to be taken in real-time to potentially circumvent pathogen spread.

### Ethiopia as a test case for real-time, gene-sequencing-based *Pst* diagnostics and surveillance

Ethiopia is the largest wheat producer in sub-Saharan Africa and is currently facing a major threat from wheat rust diseases, including yellow rust caused by *Pst*. As a potential gateway for new *Pst* strains entering Africa, it is the highest priority country in the region for rapid diagnostics [24]. In recent years, at least two novel virulent rust races have migrated into Ethiopia from other regions on prevailing winds [25]. For none of these recent incursions was it possible to obtain early, in-season detection and diagnosis of the new virulent races. Identification was possible only after disease establishment and spread had already occurred. The reliance on specialised laboratories outside of Africa for diagnosing individual *Pst* strains slows disease management decisions, a situation exacerbated by the lengthy nature of the assays, which can take many months to complete.

Currently, no developing country has the capacity to undertake real-time pathogen diagnostics on important crop diseases such as wheat yellow rust. Yet, developing countries bear the brunt of the epidemics. Therefore, we focused the deployment of our nanopore-based *Pst* genotyping system in Ethiopia. As infrastructure and logistics in developing countries can often limit the deployment of advanced diagnostic tools, we developed a mobile lab system contained in a single hard case to facilitate the movement of our MARPLE diagnostic platform between locations. Although still dependent on specialist expertise in the design phase, the resulting system itself is simple, making it highly suitable for resource-poor regions. For instance, an in-country trial illustrated that the pipeline can be used directly in Ethiopia in any lab irrespective of existing infrastructure and without the need for continuous electricity or access to additional laboratory equipment [26] (Additional file 13; Additional file 1: Table S13).

Using this platform, we determined that the Ethiopian *Pst* population structure has remained stable since 2016, with all isolates analysed being genetically closely related. As genomic-based PoC diagnostics enters the mainstream, such real-time genotyping techniques will enable rapid detection of new *Pst* strains entering East Africa. This high-resolution genetic data can then help inform deployment of *Yr* disease resistance genes to match the most prevalent races present in the region. Furthermore, such data can also be incorporated in near real-time into spatio-temporal population models for *Pst*, linking epidemiological modelling and genomic data to elucidate likely transmission events and enhance the predictive power of disease forecasting [27].

### The future of genomic-based plant pathogen diagnostics and surveillance

The utility of genomic-based approaches for real-time disease diagnostics and surveillance has been illustrated time and again during human health outbreaks. However, transferring these approaches to track fungal threats to plant health can be challenging, particularly considering their frequent obligately biotrophic nature and large genome sizes. The approach we developed herein provides a means to overcome these limitations and generate comprehensive genotypic data for pathogen strains within days of collecting material from the field, making it highly suited to disease emergencies. The mobility of our approach also obviates the movement of live samples and transfers ownership back to sample collectors in-country. In addition, such molecular-based approaches enhance our testing capacity and provide the means for rapid pre-selection of the most notable and representative isolates for complementary virulence profiling, which remains an essential but costly and time-consuming process.

One future challenge when designing similar approaches for other pathosystems will be the need for existing genomic data to define polymorphic genes for amplification. However, draft genome assemblies are available for many important fungal plant pathogens and the cost of re-sequencing diverse isolates is ever decreasing. By focusing on generating data from a small subset of genes, our approach is also relatively inexpensive and generates small, unified datasets that can then be readily explored using analytic and visualisation tools created for smaller bacterial and viral genomic datasets such as Nextstrain [28]. These tools have proved extremely informative in tracking viral pathogen evolution and spread for global human health threats [29]. Using our approach, data for plant pathogens could be incorporated immediately into such a tool to understand how disease outbreaks and novel variants spread.

## Conclusions

In this study, we developed a rapid PoC method called MARPLE diagnostics for genotyping individual *Pst* isolates directly from field-collected infected plant tissue in situ. Our targeted sequencing approach unlocks new opportunities for mobile, genomic-based, strain-level diagnostics to be applied to complex fungal pathogens. The ability to rapidly identify individual strains with specific properties such as fungicide resistance will be invaluable in guiding disease control measures and represents a new paradigm for approaches to tracking plant disease.

## Methods

### RNA extraction and RNA-seq of global *Pst*-infected plant samples

A total of 100 *Pst*-infected wheat samples were collected from 2015 to 2017 from nine countries and stored in the nucleic acid stabilisation solution RNAlater® (Thermo Fisher Scientific, Paisley, UK). RNA was extracted using a Qiagen RNeasy Mini Kit following the manufacturer’s instructions (Qiagen, Manchester, UK), with the quality and quantity of RNA assessed using an Agilent 2100 Bioanalyzer (Agilent Technologies, CA, USA). cDNA libraries were prepared using an Illumina TruSeq RNA Sample Preparation Kit (Illumina, CA, USA) and sequenced on the Illumina HiSeq 2500 platform at GENEWIZ (NJ, USA). Adaptor and barcode trimming and quality filtering were performed using the FASTX-Toolkit (version 0.0.13.2). Paired-end reads (101 bp) were aligned to the PST-130 reference genome [13], and single nucleotide polymorphism (SNP) calling was performed as described previously [11].

### Phylogenetic analysis

All phylogenetic analyses were carried out using a maximum-likelihood approach with RAxML 8.0.20 using the GTRGAMMA model, with 100 replicates using the rapid bootstrap algorithm [30]. For analysis of the global *Pst* population, nucleotide residues were filtered using a minimum of 20× depth of coverage for sites that differed from the PST-130 reference genome [13] and 2x coverage for sites that were identical. These filtered positions were then used to independently generate consensus gene sets that incorporated separately the SNPs identified within the gene space for each *Pst* isolate as described previously [31]. The third codon position of these genes was used for phylogenetic analysis. For samples sequenced on the MinION platform, the 242 polymorphic *Pst* genes were utilised for phylogenetic analysis. All phylogenetic trees were visualised in Dendroscope version 3.5.9 [32] or MEGA version 7 [33].

### Population structure analysis of global *Pst* isolates

The genetic subdivision of the 301 global *Pst* isolates was assessed using nonparametric multivariate clustering without any predetermined genetic model. This method was selected to avoid bias associated with providing location information of *Pst* isolates from different lineages to the model. First, biallelic SNP sites introducing a synonymous change in at least one isolate were selected and extracted for all 301 *Pst* isolates. These data were used for multivariate analysis using DAPC implemented in the Adegenet package version 2.1.1 in the R environment [14]. The number of population clusters (Kmax) was identified using the Bayesian Information Criterion (BIC). After initially selecting five genetic groups, DAPC was repeated for isolates within each of these population clusters to define subdivisions within each group.

### Selection of highly polymorphic *Pst* genes

To select a polymorphic *Pst* gene set that could be used to accurately reconstruct the *Pst* global phylogeny, alignments of sequences from the 301 *Pst* global isolates against the PST-130 reference genome [13] were filtered for sites represented in at least 60% of the isolates. Next, *Pst* isolates which had at least 60% of the sites represented at 20× coverage were selected. For each position in the alignment, the degree of polymorphism was determined by calculating the number of unique bases found in a given position in each of the 301 *Pst* global isolates. This number was then divided by the length of the gene to calculate the number of SNPs per kilobase for each gene (SNPs/kb). All genes within a range of 1–3 kb that met a minimum SNPs/kb value threshold were then aggregated to select 1690, 1006, 748, 500, 402, 301, 204, 151 and 100 of the most polymorphic genes using progressively increasing SNPs/kb cut-off values (0.001, 0.006, 0.0105, 0.018, 0.023, 0.0285, 0.036, 0.042 and 0.051, respectively) and used to carry out phylogenetic analysis as described previously. To calculate the number of SNPs present in each of the five global groups defined by DAPC analysis, concatenated alignments of the 242 polymorphic *Pst* genes for each of the 301 global *Pst* isolates were used to calculate the total number of SNPs present in each sample using SNP-sites [34] and plotted using the ggplot2 package [35] in R.

### Annotation of the polymorphic *Pst* gene set

The genomic location of each of the 242 polymorphic *Pst* genes was identified by mapping these gene sequences to the *Pst*-104 genome [16] using minimap2 version 2.15 [17] with parameters recommended in the manual for pairwise genome alignment (minimap -ax asm10). Locations were processed into BED format using bedtools version 2.27.0 [36] and analysed and plotted using R. GO term analysis of the 242 genes was conducted using BLAST2GO version 5.2 [37].

### DNA extraction and amplification of *Pst* genes

*Pst*-infected wheat leaf samples were collected from the field and stored in RNAlater®. These samples consisted of a single lesion or rust pustule. Excess RNAlater® was removed, and ~ 10–20 mg of tissue was used for each DNA extraction. DNA was extracted using a DNeasy 96 Plant Kit (Qiagen, Manchester, UK) following the manufacturer’s instructions and eluted twice through the column in a total of 30 μl elution buffer. The DNA extracted was used for amplifying the 242 variable *Pst* genes via PCR with four pools containing oligonucleotides (primers) with different concentrations optimised for multiplex PCR (Additional file 1: Table S4) using Q5® Hot Start High-Fidelity 2X Master Mix (New England Biolabs, MA, USA). PCR conditions used were 98 °C for 30 s, 40 cycles of 98 °C for 10 s, 63 °C for 30 s and 72 °C for 2 min 30 s, and a final extension of 72 °C for 2 min. PCR products were purified using a QIAquick PCR Purification Kit (Qiagen, Manchester, UK) following the manufacturer’s instructions and eluted twice through the column in a total of 30 μl elution buffer. The concentration of purified PCR products from each primer pool was measured using a Qubit dsDNA HS Assay Kit (Invitrogen, MA, USA) following the manufacturer’s instructions.

### Illumina library preparation for amplicon sequencing

Four *Pst*-infected wheat samples (17.0504, 17.0505, 17.0506 and 17.0507) were utilised for amplicon sequencing using the MiSeq platform (Illumina, CA, USA). Following DNA extraction and PCR amplification of the 242 selected *Pst* genes, an equal mass of purified PCR products from each of the four primer pools was combined prior to library preparation, giving a total of 1 μg DNA (250 ng per primer pool; Additional file 1: Table S14). Samples were prepared for sequencing using a KAPA HyperPlus Library Preparation Kit (Roche, Basel, Switzerland) following the manufacturer’s instructions. PCR products were fragmented enzymatically into sizes of approximately 600 bp using a reaction time of 10 min. Each sample was tagged with a unique barcode to enable sample identification. The resulting libraries had insert sizes of 790–911 bp and were made into an equimolar pool of 40 μl prior to sequencing (Additional file 1: Table S14). Libraries were sequenced using an Illumina MiSeq platform and MiSeq Reagent Kit v3 150 cycles (Illumina, CA, USA) following the manufacturer’s instructions.

### MinION sequencing of Ethiopian *Pst*-infected wheat samples

For each of the 51 *Pst*-infected wheat samples collected in Ethiopia in 2016 (one sample) and 2017 (50 samples), an equal mass of PCR products from each of the four primer pools was combined prior to library preparation with a total of between 16 and 400 ng amplicon DNA (4–100 ng per primer pool; Additional file 1: Table S15). Samples were then processed into multiplexed libraries containing eight samples each using a PCR Barcoding Kit, SQK-PBK004 (Oxford Nanopore Technologies, Oxford, UK) following the manufacturer’s instructions. Equimolar pools were made using eight samples having different barcode tags with a total mass of DNA between 10 and 1000 ng (1.3–100 ng per sample; Additional file 1: Table S15). Pooled samples were sequenced on a MinION sequencer using Flow Cells FLO-MIN106D R9 version or FLO-MIN107 R9 version (Oxford Nanopore Technologies, Oxford, UK) following the manufacturer’s instructions until 2 million reads were generated (250,000 per sample; Additional file 1: Table S15).

### In-field sequencing of *Pst*-infected wheat samples in Ethiopia

Four *Pst*-infected wheat leaf samples (Et-0001, Et-0002, Et-0003, Et-0004) were collected from different locations in Ethiopia in 2018 (Additional file 1: Table S1) and stored in RNAlater®; approximately 10–20 mg of tissue was used for DNA extraction. Samples were disrupted in 200 μl lysis buffer [0.1 M Tris-HCl pH 7.5, 0.05 M ethylenediaminetetraacetic acid (EDTA) pH 8 and 1.25% sodium dodecyl sulphate (SDS)] using a micropestle for approximately 30 s. The ground tissue was allowed to settle and the supernatant removed. DNA was purified from the supernatant by adding 200 μl AMPure XP beads (Beckman Coulter, CA, USA) to each sample, mixing briefly and incubating at room temperature for 15 min. Tubes were placed on a magnetic rack to allow the supernatant to clear. The supernatant was removed and discarded before beads were washed twice with 80% ethanol and the supernatant removed. The beads were left on the magnetic rack to dry, and 30 μl nuclease-free water was added to resuspend the pellet. Tubes were removed from the magnet and mixed before incubation at room temperature for 2 min. The tubes were incubated briefly on the magnetic rack, and the clear supernatant containing DNA was transferred into a new tube. The extracted DNA was used for amplifying the 242 variable *Pst* genes via PCR with four pools containing primers with different concentrations optimised for multiplex PCR (Additional file 1: Table S4) using AmpliTaq Gold™ 360 Master Mix (Applied Biosystems, CA, USA) in a 50 μl reaction volume. The PCR conditions used were 95 °C for 10 min, 40 cycles of 95 °C for 15 s, 51 °C for 30 s and 72 °C for 4 min, and a final extension of 72 °C for 7 min. DNA was purified from the PCR product using 50 μl AMPure XP beads (Beckman Coulter, CA, USA). For each sample, an equal volume of each purified PCR pool was combined for each library preparation. The final volume per sample entered into each library preparation was 7.5 μl (1.88 μl per purified PCR pool). Samples were prepared for sequencing using a Rapid Barcoding Kit, SQK-RBK004 (Oxford Nanopore Technologies, Oxford, UK). Libraries were sequenced on the MinION platform using Flow Cells FLO-MIN106D R9 version (Oxford Nanopore Technologies, Oxford, UK) following the manufacturer’s instructions until 250,000 reads were generated (Additional file 1: Table S15).

### Data analysis of samples sequenced using the MinION platform

Following base calling and demultiplexing using Albacore version 2.3.3 (Oxford Nanopore Technologies, Oxford, UK), reads from each sample generated on the MinION platform were trimmed using porechop version 0.2.3 (https://github.com/rrwick/Porechop) and aligned to the 242-gene set from PST-130 using BWA-MEM version 0.7.17 [38] with default settings and processed using SAMTOOLS version 1.8 [39]. Oxford nanopore is known to be error prone and hence BWA-MEM was selected as it is particularly suited to such datasets. Consensus sequences based on these alignments were generated for each sample by calling bases with a minimum of 20× coverage. Heterokaryotic positions were deemed as such when the minor allele had a minimum allele frequency of at least 0.25. For phylogenetic analysis, concatenated alignments of the 242-gene set from each of the *Pst* samples were used.

### Comparative analysis of the Illumina MiSeq and MinION sequencing platforms

Four samples (17.0504, 17.0505, 17.0506 and 17.0507) were sequenced on the MinION and the Illumina MiSeq platforms as described above. Data generated on the MinION platform were analysed as described. The MiSeq data were aligned to the 242 *Pst* gene set using BWA-MEM version 0.7.17 [38] with default settings and processed using SAMTOOLS version 1.8 [39]. Consensus sequences based on these alignments were generated for each sample by calling bases with a minimum of 20x coverage. Heterozygous positions were deemed as such when the minor allele had a minimum allele frequency of at least 0.25. To compare the MinION and MiSeq platforms, the above procedure of generating MinION consensus sequences was repeated using different coverage cut-off values and the sequences for each of the 242 *Pst* genes at each of the different coverage cut-off values were compared against the Illumina consensus sequence (called using a 20× coverage cut-off). Positions that were deemed ambiguous (< 20× coverage) in the MiSeq consensus sequences were excluded from the analysis. Percentage identity between the MinION and MiSeq consensus sequences was calculated using the ggplot2 package in R [35]. The coverage values for each gene as a percentage of the total coverage for each of the four samples sequenced using the MiniON and MiSeq platforms was calculated using SAMTOOLS version 1.8 [39] and R. A heatmap of the number of SNPs found in each of the 242 genes for each of the four samples compared with the PST-130 reference genome using Illumina MiSeq and MinION sequencing technologies was generated using the pheatmap package in R [40].

## Additional files

Additional file 1 Tables S1-S15. Microsoft Excel Workbook containing 15 worksheets. Table S1: Description of *Pst* isolates. Table S2: Number of reads aligned to the PST-130 reference genome for the 100 RNA-seq datasets. Table S3: Number of reads aligned to the PST-130 reference genome for the 25 genomic datasets utilised herein. Table S4: Oligonucleotide (primer) sequences and pooling strategy for the 242 *Pst* genes selected. Table S5: Genomic location and functional annotation of the 242 *Pst* genes selected. Table S6: Number of reads aligned to the PST-130 reference genome for the 4 MiSeq amplicon datasets. Table S7: Number of reads generated on the MinION platform that aligned to the 242 *Pst* genes selected. Table S8: Time taken to generate a given number of MinION reads for the four Ethiopian *Pst*-infected wheat samples. Table S9: Details of SNP positions per gene detected in MinION and Illumina datasets generated from four *Pst* isolates. Table S10: Details of homokaryotic and heterokaryotic positions in consensus sequences obtained from Illumina MiSeq and MinION platforms. Table S11: Number of reads aligned to the PST-130 reference genome for the Ethiopian *Pst* RNA-seq datasets. Table S12: Additional *Pst* reference isolates representing diverse virulence phenotypes and key genetic groups of a worldwide phylogeny of *Pst*. Table S13: Components used in the MARPLE pipeline. Table S14: Details of Illumina library preparation of 4 *Pst*-infected samples. Table S15: Details of libraries made for MinION sequencing of *Pst*-infected wheat leaf samples. (XLSX 4138 kb)

Additional file 2 Phylogenetic analysis of the 301 global *Pst* isolates. Newick format. (NEWICK 16 kb)

Additional file 3 Phylogenetic analysis of the 301 global *Pst* isolates with bootstrap values. Newick format. (NEWICK 16 kb)

Additional file 4 Multivariate discriminant analysis of principal components (DAPC) performed on the 301 global *Pst* isolates**. (a)** The Bayesian information criterion (BIC) was calculated for combined DAPC analysis of all 301 global *Pst* isolates, which indicated an optimal clustering solution of K = 5. **(b-f)** Further DAPC analysis was carried on each of the initial five population clusters, and assessment of the BIC was used to determine the optimal clustering solution (red circle). The *Y*-axis corresponds to the BIC, a goodness-of-fit measurement calculated for each K value. (EPS 994 kb)

Additional file 5 A minimum of 100 *Pst* genes is sufficient to accurately reconstruct the global phylogeny**.** Phylogenetic trees were generated with 1690, 1006, 748, 500, 402, 301, 204, 151 and 100 of the most polymorphic genes using a maximum-likelihood approach. Colours represent *Pst* isolates from similar geographical locations and/or genetic backgrounds. (EPS 2671 kb)

Additional file 6 Phylogenetic analysis of the 301 global *Pst* isolates using sequences for the 242 selected genes**.** Newick format. (NEWICK 16 kb)

Additional file 7 Phylogenetic analysis of the 301 global *Pst* isolates using sequences for the 242 selected genes, with bootstrap values. Newick format. (NEWICK 17 kb)

Additional file 8 RNA-seq analysis of the 51 Ethiopian *Pst* isolates using the 242 genes defined herein shows they are genetically closely related. Phylogenetic analysis was carried out using the 51 *Pst* isolates collected in Ethiopia in the 2017/2018 growing season and the 301 global *Pst* isolates, using a maximum-likelihood model and 100 bootstraps. The 51 Ethiopian *Pst* isolates grouped in two closely related clades. One of the two clades also grouped closely with *Pst* isolates from genetic group 2, however, this was supported by a low bootstrap value that may be indicative of uncertainty in the grouping of these isolates (Additional file 12). Scale indicates the mean number of nucleotide substitutions per site. (EPS 1459 kb)

Additional file 9 Phylogenetic analysis for the 301 global *Pst* isolates, 55 Ethiopian *Pst*-infected field samples and *Pst* isolates representative of several major genetic groups. Data for the Ethiopian *Pst* isolates and representative genetic group isolates were generated on the MinION platform. Newick format. (NEWICK 20 kb)

Additional file 10 Phylogenetic analysis for the 301 global *Pst* isolates, 55 Ethiopian *Pst*-infected field samples and *Pst* isolates representative of several major genetic groups, with bootstrap values. Data for the Ethiopian *Pst* isolates and representative genetic group isolates were generated on the MinION platform. Newick format. (NEWICK 21 kb)

Additional file 11 Phylogenetic analysis of the 301 global *Pst* isolates using sequences for the 242 genes selected and data for these genes generated on the HiSeq platform for the 51 Ethiopian *Pst* isolates**.** Newick format. (NEWICK 19 kb)

Additional file 12 Phylogenetic analysis of the 301 global *Pst* isolates using sequences for the 242 genes selected and data for these genes generated on the HiSeq platform for the 51 Ethiopian *Pst* isolates with bootstrap values. Newick format. (NEWICK 20 kb)

Additional file 13 Detailed illustration of the full MARPLE pipeline**.** The complete list of steps in the Mobile And Real-time PLant disEase (MARPLE) diagnostics pipeline (A-Q). (EPS 1525 kb)

## Data Availability

The raw transcriptomic and genomic sequence data that support the findings of this study have been deposited in the European Nucleotide Archive (ENA: ERP113880) [41]. All custom computer code and an easy-to-follow guide for installing prerequisite software using the Python Conda package manager has been deposited on github (https://github.com/SaundersLab/MARPLE_plant_pathogen_diagnostics) [42].
